# Genetic Characterization of the Immortalized Human Nasopharyngeal Carcinoma Cell Line NPC/HK1

**DOI:** 10.1002/cam4.70422

**Published:** 2025-02-04

**Authors:** Anna Makowska, Udo Kontny, Josef van Helden, Barbara Hildebrandt, Herdit M. Schüler, Ralf Weiskirchen

**Affiliations:** ^1^ Division of Pediatric Hematology, Oncology and Stem Cell Transplantation University Hospital Aachen Aachen Germany; ^2^ Institute of Molecular Pathobiochemistry, Experimental Gene Therapy and Clinical Chemistry (IFMPEGKC) University Hospital Aachen Aachen Germany; ^3^ Institute of Human Genetics Medical Faculty and University Hospital Düsseldorf, Heinrich Heine University Düsseldorf Germany

**Keywords:** cell authentication, CLASTR, Epstein–Barr virus, ICLAC, karyotyping, NPC, STR

## Abstract

**Background:**

Human nasopharyngeal carcinoma (NPC) cell lines are in vitro model systems that are widely available, easy to handle, and provide an unlimited supply of material. They also bypass ethical concerns associated with the use of primary human cells or tissue. However, many of these cell lines including 5‐8F, 6‐10B, CNE‐1, CNE‐2, HNE‐1, HONE‐1, SUNE1, SUNE2, and NPC‐TW01 have been shown to be misidentified or cross‐contaminated. While simple molecular genotyping techniques such as short tandem repeat profiling of human cell lines are available to confirm cell line identity, scientists often do not implement strategies to avoid misidentification. This has resulted in a large volume of publications containing incorrect information.

**Methods:**

In this paper, we have established a cell line karyogram that contains several marker chromosomes and a set of typical aberrations characteristic of NPC/HK1.

**Results and Conclusions:**

Combined with the typical multiloci short tandem repeat signature of NPC/HK1, the cytogenetic analysis provides an effective means to avoid unreliable experimental outcomes and scientific misinterpretation.

## Graphical abstract

Immortalized nasopharyngeal carcinoma cell lines in biomedical research.

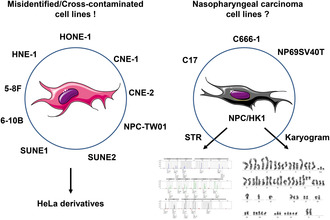

## 1 Introduction

Nasopharyngeal carcinoma (NPC) is a type of head and neck cancer that occurs in almost all parts of the world, with the majority of cases found in South East Asia. It is well‐known that Epstein–Barr virus (EBV) infection is crucial for NPC tumorigenesis. Over the last few decades, more than 80 immortalized NPC cell lines have been established, serving as valuable tools for research in NPC. However, it has been discovered that many of these cell lines are misidentified or contaminated by other cells such as HeLa [1, 2]. Unfortunately, some prominent cell lines including 5‐8F, 6‐10B, CNE‐1, CNE‐2, HNE‐1, HONE‐1, SUNE1, SUNE2, and NPC‐TW01 are still widely used in many laboratories investigating the aspects of NPC biology [1, 2, 3].

Short tandem repeat (STR) testing is currently considered the standard method for cell line authentication due to its accuracy, speed, reliability, and cost‐effectiveness. Additionally, defining a consistent karyotype or identifying specific numerical and structural abnormalities can serve as reliable markers to authenticate a specific cell line.

In this report, we present the G‐banded karyotype of NPC/HK1, a cell line originally established over four decades ago in China from a tumor tissue outgrowth of a late NPC relapse in a 60‐year‐old Chinese male who had been initially diagnosed with a poorly differentiated squamous carcinoma at the age of 41 [4]. We further confirmed the original finding that this cell line is negative for EBV [4] and verified the STR profile for this cell line previously reported [5]. Furthermore, we demonstrate that the STR profile is specific and distinguishable from misidentified NPC cell lines HNE‐1, HONE‐1, HONE‐1/AKATA, CNE‐1, CNE‐2, and NPC‐TW01, as well as cell lines C17, C666‐1, and NP69SV40T, which are supposed to be of NPC origin.

## 2 Materials and Methods

### 2.1 Cell Sources

Cell lines CNE‐2 and C17 were generously provided by Prof. Pierre Busson at the Gustave Roussy Institute in Paris, France. The cell line NPC/HK1 was obtained from Prof. Lo Kwok Wai at the Chinese University of Hong Kong, China. Cell lines HONE‐1, HONE‐1/AKATA, and the nasopharyngeal epithelial cell line NP69SV40T established by the immortalization of biopsy‐derived primary nasopharyngeal epithelial cells with SV40T were supplied by Prof. George Tsao^†^ at the Chinese University of Hong Kong. NPC‐TW01 was supplied by Prof. Chin‐Tarng Lin at the National Taiwan University Hospital. Cell lines CNE‐1 and C666‐1 were kindly provided by Prof. Fei‐Fei Liu at the University of Toronto, Canada. HNE‐1 was obtained from Prof. Qian Tao at the Chinese University of Hong Kong. All cell lines used in this study have been authenticated using STR profiling.

### 2.2 Cell Culture

The EBV‐negative cell lines NPC/HK1 [4], CNE‐1 [6], CNE‐2 [7], HNE‐1 [8], HONE‐1 [8], and NPC‐TW01 [9] were cultured in Dulbecco's modified Eagle's medium (#P04‐04510, PAN Biotech, Dorset, UK). The EBV‐positive cell lines C17 [10] and C666‐1 [11] were maintained in RPMI1640 medium (#21875‐091, Gibco, Paisley, UK). The nasopharyngeal epithelium cell line NP69SV40T [12] was cultured in a keratinocyte‐serum‐free medium (#17005‐042, Gibco; NY, USA). All media were supplemented with 10% fetal bovine serum (#10500‐064, Gibco, Paisley, UK), 100 U/mL penicillin, and 100 mg/mL streptomycin (#15140‐122, Gibco, NY, USA). Additionally, RPMI1640 medium for the C17 cell line included the Rho‐associated coiled‐coil containing the kinase inhibitor (Y‐27632) (#S1049, Selleckchem, Munich, Germany). Cells were incubated in a humidified environment with 95% air and 5% CO_2_ at 37°C. Medium exchange was conducted every second day, and cells were subcultured using 0.05% Trypsin EDTA solution (#P10‐0231SP, PAN Biotech, Aidenbach, Germany).

### 2.3 Preparation of NPC/HK1 Metaphase Chromosomes and Karyotyping

The preparation of chromosomes for NPC/HK1 was conducted using standard procedures. To summarize, semiconfluent NPC/HK1 cultures were exposed to a colcemid solution (Gibco, ThermoFisher Scientific, Dreieich, Germany), detached, and then harvested through centrifugation. Subsequently, the cells were treated with a 0.56% (*w*/*v*) hypotonic potassium chloride solution and fixed using a mixture of methanol and acetic acid (3:1). Chromosome spreads were air‐dried, slides were treated with a 0.025% (*w*/*v*) trypsin solution, and finally stained with Giemsa solution to visualize the G‐banding pattern.

### 2.4 Short Tandem Repeat Profiling

The STR profiling and interspecies contamination tests for the different cell lines used in this study were conducted using the cell line authentication service provided by IDEXX BioAnalytics (Kornwestheim, Germany). They utilized the CellCheck Human system, which consists of 16 species‐specific STR markers. Eight of these markers are based on the recommendations of established standard guidelines from the American Tissue Culture Collection (ATCC), which suggest a minimum of eight core STR loci (CSF1PO, D13S317, D16S539, D5S818, D7S820, TH01, TPOX, and vWA), along with the Amelogenin (AMEL) gene for gender identification [13]. Additionally, the CellCheck16 Human system contains seven additional markers (D18S51, D21S11, D3S1358, D8S1179, FGA, Penta D, and Penta E), increasing the significance of the test.

### 2.5 STR Similarity Search

All STR similarity searches were conducted using the Cellosaurus STR Similarity Search Tool CLASTR 1.4.4 (release 41.0) [14]. Additional information about the Cellosaurus STR database can be found elsewhere [15, 16]. The search settings for each case were as follows: Scoring algorithm—Tanabe; Mode—nonempty markers; Score filter—60%; Min. Markers—8, and Max. Results—200.

### 2.6 EBV Detection by Copy Number Assay

The EBV load and copy number were detected in cell supernatants using the Panther Fusion EBV Quant test kit (# AM‐26019‐801, Hologic Deutschland GmbH, Wiesbaden, Germany) on a fully automated molecular Panther analyzer. This analyzer performed all steps, from primary sample handling to DNA extraction and real‐time PCR amplification without manual intervention. The assay is standardized to the first international WHO standard (NIBSC code: 09/260) for EBV [17] and has robust coverage with a limit of detection (LoD) of 54.1 IU/mL in plasma and 200.9 IU/mL in whole blood. In our measurements, the EBV titers measured in cell supernatants were reported as copies/mL and classified as positive when the test indicated a viral load higher than 230 copies per mL, estimating a conversion factor for EBV of approximately 1 IU/mL = 4.255 copies/mL when assuming the complexity of the culture medium to be similar to plasma. The threshold was determined as part of the test validations specified by the accreditation through dilution and spiking experiments.

## 3 Results

**FIGURE 1:**
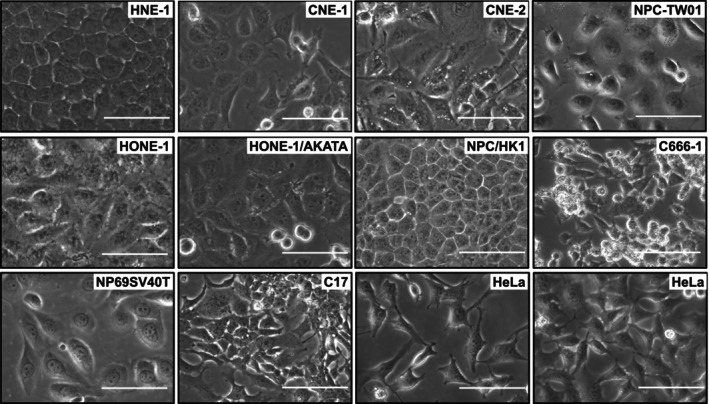
Phenotypic characteristics of cell lines commonly used in research on NPC. The cell lines HNE‐1, CNE‐1, CNE‐2, NPC‐TW01, HONE‐1, HONE‐1/AKATA, NPC/HK1, C666‐1, NP69SV40T, and C17 are immortalized cell lines used in biomedical research. While these cell lines have distinct phenotypic appearances, there is a debate regarding whether some of these cell lines are misidentified cells originating from HeLa or derivatives thereof. For comparison, images of HeLa cells at low and high densities are included. The cell images were captured at a magnification of 400×, with scale bars representing 100 μm.

The cell line NPC/HK1 was established as an outgrowth from a tumor tissue explant of a 60‐year‐old Chinese male who had a poorly differentiated squamous carcinoma at the age of 41. The line was presented to the public as a stable, EBV‐negative cell line after 72 passages that had been successfully achieved over a period of approximately 1 year [4]. Since then, the cell line has been used as an experimental tool in many studies. Typically, the cells grow polygonal in shape and distribute in a mosaic pattern with many round cells with a doubling time of 28 ± 2 h [4]. These phenotypic characteristics are stable, even after over four decades (Figure 1).

It has been well‐recognized for many years that a number of NPC cells are contaminated with HeLa [1, 5]. Nevertheless, many of these cell lines are still widely used in NPC research [1]. Therefore, we first performed STR profiling for NPC/HK1 to confirm that the line we have in our laboratory is indeed NPC/HK1. Using the CellCheck Human system for STR profiling, which consists of 16 species‐specific STR markers AMEL, CSF1PO, D13S317, D16S539, D5S818, D7S820, TH01, TPOX, vWA, D18S51, D21S11, D3S1358, D8S1179, FGA, Penta D, and Penta E, we were able to demonstrate that the 16 structural variant sites are in full agreement with the previously reported one [1, 5]. This is not surprising because we obtained the cell line from the laboratory that first established the STR profile for this cell line.

**FIGURE 2:**
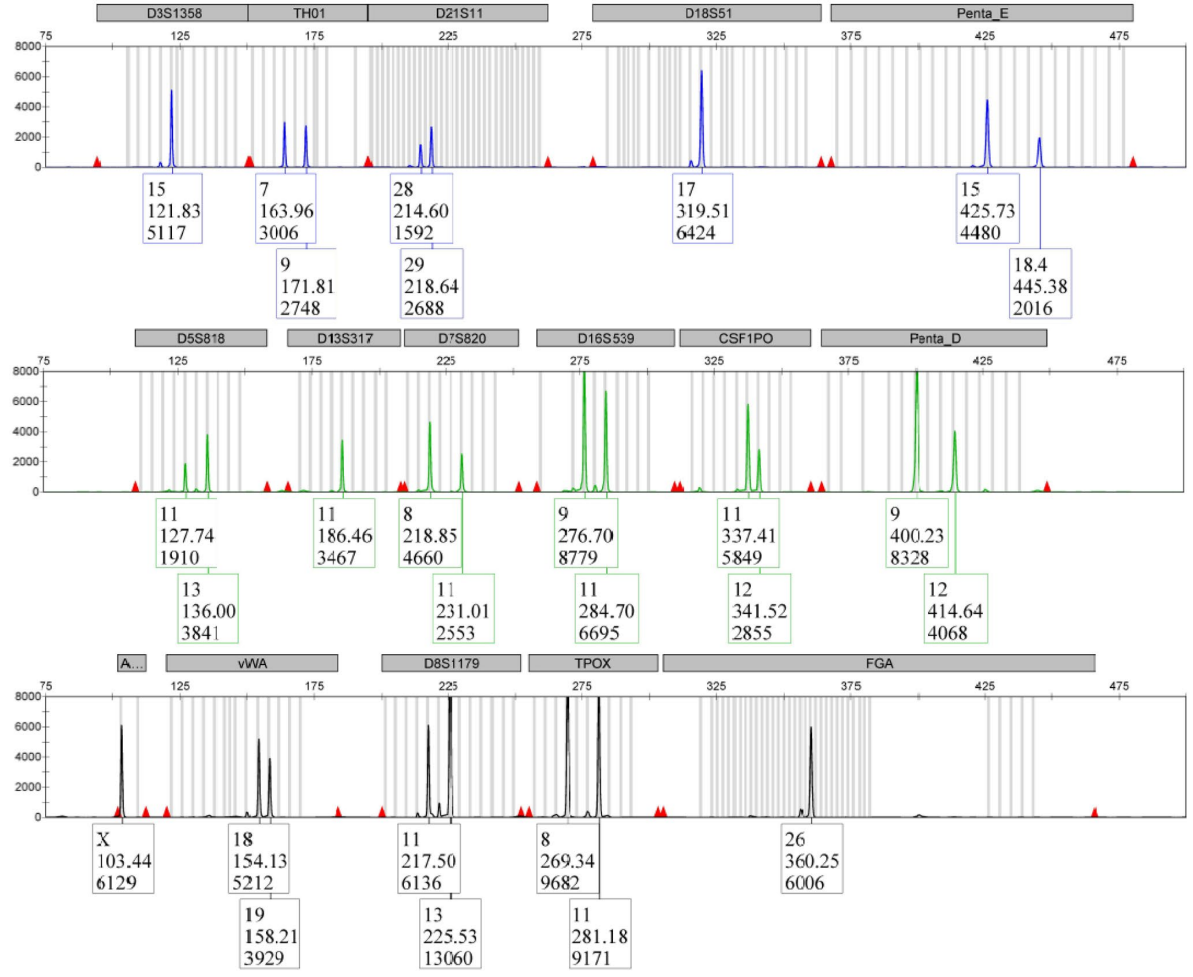
Short tandem repeat (STR) profiling of NPC/HK1 cells. Genomic DNA from NPC/HK1 was profiled with the CellCheck Human STR system that contains 16 species‐specific markers. The profile of the 16 markers suggests a unique STR profile.

The respective STR profile was absolutely unambiguous (Figure 2) and completely different from that of HeLa and the misidentified cell lines HNE‐1, HONE‐1, HONE‐1/AKATA, CNE‐1, CNE‐2, and NPC‐TW01 (Figure S1). Moreover, the STR profile was different from that of C17, C666‐1, and NP69SV40T (Figure S2 and Table 1). Importantly, an STR similarity search conducted using the Cellosaurus STR Similarity Search Tool CLASTR 1.4.4 (release 41.0) further demonstrated that the profile is unique. The highest homologies existed with the neuroblastoma cell line CHLA‐143 (CVCL_6593; 65.22%), the cecum adenocarcinoma cell line NCI‐H498 (CVCL_1563, 61.20%), the primitive neuroectodermal tumor line CHLA‐57 (CVCL_0B49, 60.80%), the monocytic leukemia cell line NOMO‐1 (CVCL_1609, 60.70%), the embryonic stem cell chHES‐185 (CVCL_A921, 60.30%), and the squamous cell carcinoma cell line UPCI‐SCC‐032 (CVCL_C029, 60.00%), respectively (Table 2).

**TABLE 1:** Short tandem repeat profiling in representative cell lines used in NPC research using the CellCheck Human system.

Cell line	AMEL	CSF1PO	D3S1358	D5S818	D7S820	D8S1179	D13S317	D16S539	D18S51	D21S11	FGA	PentaD	Penta E	TH01	TPOX	vWA
NPC/HK1	X	11,12	15	11,13	8,11	11,13	11	9,11	17	28,29	26	9,12	15,18.4	7,9	8,11	18,19
NPC‐TW01	X	10,11	15,18	11,12	10,12	12,16	10,12,13.3	9,10	13,16	27,30	21	9,12	17,20	6,7,9	8,12	14,16,(17)^a^
HNE‐1	X	10,11	15,18	11,12	10,12	12,16	10,12,13.3	9,10	13,16	27,30	18,21	9,12	17,20	6,7,9	8,12	14,16
HONE‐1	X	10,11	15,16,18	11,12	10,12	12,16	10,12,13.3	9,10,11	13,16	27,30	18,21	9,12	17,20	6,7,9	8,12	14,16
HONE1/AKATA	X	10,11	15,18	11,12	10,12	12,16	10,12,13.3	9,10	13,16	27,30,31	18,21	9,12	17,20	6,7,9	8,12	14,16
CNE‐1	X	10,11	15,18	11,12	10,12	12,17	10,12,13.3	9,10	13,16	27,30	21	9,12	17	7,9	8,9,12	14,16
CNE‐2	X	10,11	15,18	11,12	10,12	12,17	10,12,13.3	9,10	13,16	27,30	21	9,12	17	7,9	8,9,12	14,16
C666‐1	X,Y	11,15,16	16,17	11	11,12	11,12,15,16	8,11	10	16	28,29,31.2	24	9,10	11,15	6,8	8,11	17,18
C17	X,Y	10	15	11,14	11	11,15	11	12	20	30	23	12	15,22	6	9	14,15
NP69SV40T	X,Y	12,13	17	11	11	15,16	10,12	11,12	13	31	21,22	8	11,16	7	11	16,19
HeLa	X	9,10	15,18	11,12	8,12	12	12,13.3	9,10	16	27,28	18,(19),21	8,15	7,17	7	12	16,18

^a^ The structural variant 17 in vWA was lost at later passage numbers in NPC‐TW01. While the STR profile at this variant site was found to be tri‐allelic at passage number 3, the variant allele 17 was not detected in cells at later passage numbers (> 18). Note that these passage numbers refer to those in our laboratory.

**TABLE 2:** Matching of the 16 short tandem repeats obtained for NPC/HK1 cells to known STR profiles listed in the Cellosaurus database.^a^

Accession^a^	Name	Score, (%)	AMEL	CSF1PO	D3S1358	D5S818	D7S820	D8S1179	D13S317	D16S539	D18S51	D21S11	FGA	PentaD	Penta E	TH01	TPOX	vWA
	NPC/HK1	NN	X	11,12	15	11,13	8,11	11,13	11	9,11	17	28,29	26	9,12	15,18.4	7,9	8,11	18,19
CVCL_7084	NPC/HK1	100.00	X	11,12	15	11,13	8,11	11,13	11	9,11	17	28,29	26	ND	ND	7,9	8,11	18,19
CVCL_6593	CHLA‐143	65.22	X	11,12	15	12,13	9,11	12,13	12,13	9,11	15,17	29,30	22,25	ND	ND	7,9	11	18,19
CVCL_1563	NCI‐H498	61.20	X	12	15	13	8,11	11,13	9,11	9,11	15	29,31	22,24	9,10	5,15	7	8	15
CVCL_0B49	CHLA‐57	60.80	X	10,12	15	11,13	12,13	11,12	11,12	10,11	17	28,29	20,25	ND	ND	7,9	8,11	16,17
CVCL_1609 Best^b^	NOMO‐1	60.70	X	11,12	15,17	11,13	8,10	13,14	8,10	9,11	17,21.1	29,32.2	21,22	9,12	15,17	6,9	8,12	17,18
CVCL_1609 Worst^b^	NOMO‐1	60.70	X	11,12	15,16	11,13	8,10	13,14	8,10	9,11	17,22	29,32.2	21,22	9,12	15,17	6,9	8,12	17,18
CVCL_A921	chHES‐185	60.30	X,Y	11	15,16	11,13	8,11	13,14	10,11	11	14,16	29,32.2	23.2,26	9,12	11,17	7,9	8	14,17
CVCL_C029	UPCI‐SCC‐032	60.00	X	11	15	13	11,14	13	8,11	9,13	17	29,31	22,22.2	11,12	12,15	7,9	8,11	16

^a^ The search was conducted in the Cellosaurus release 49.0 at May 28, 2021 containing 8701 human cell lines with STR profiles.

^b^ Note that in the case of NOMO‐1 (CVCL_1609), two slightly different STR profiles were reported resulting in best/worst similarity scores. In this table, only cell lines' with a similarity score of at least 60% are listed.

Similar to HeLa and many other cell lines that are supposed to be of NPC origin (NP69SV40T, HNE‐1, HONE‐1, NPC‐TW01, and CNE‐2), the cell line NPC/HK1 tested negative for EBV, while the supernatants of other cultured NPC cell lines (C666‐1, C17, and HONE‐1/AKATA) showed a high viral load (C666‐1: 1,040,000 copies/mL; C17: 586,000 copies/mL; HONE‐1/AKATA: 1,990,000 copies/mL).

In the initial study, the authors mentioned both numerical and structural chromosomal aberrations including fragmentation, breakage, minute bodies, dicentric, giant acrocentric, and giant submetacentric chromosomes [5]. Individual cells showed chromosome numbers ranging from 55 to over 100 chromosomes. However, a more detailed karyotype describing the amount of chromosome count and morphology of NPC/HK1 had not been established yet. Therefore, we performed karyotyping of this cell line.

We identified a near‐triploid complex altered karyotype in 25 mitoses of the cell line NPC/HK‐1 with 66–71 chromosomes present. The third gonosome was missing, leading us to suspect the loss of a Y chromosome, as the tumor tissue originated from a male patient.

**FIGURE 3:**
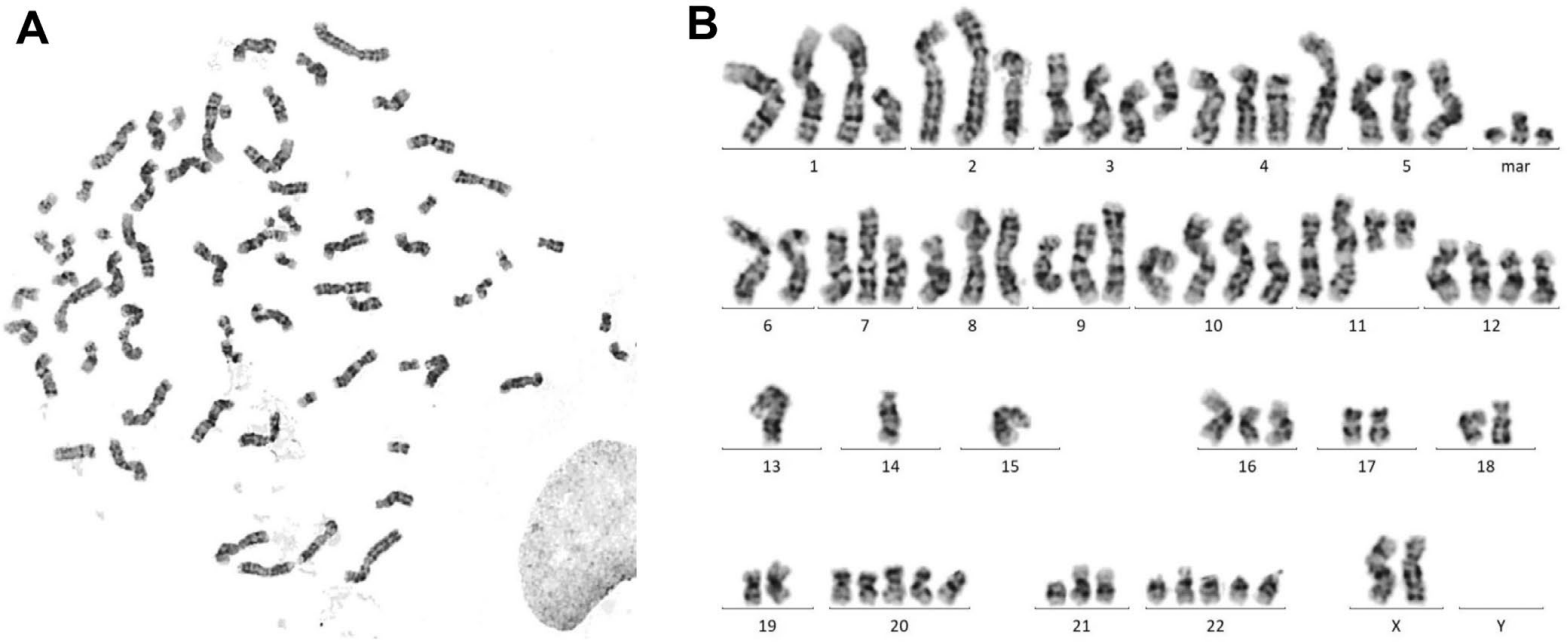
Karyogram and short tandem repeat profile of NPC/HK1 cells after Giemsa stain in light microscopic analysis. (A) Mitotic spread of NPC/HK1 cells. (B) Representative karyogram of NPC/HK1 cells showing the following aberrations: 66‐71,XX,‐Y, +del(1)(p11),del(2)(q23),del(3)(p13),+del(3)(q12),del(4)(p15), +add(4)(p14), add(5)(p14),‐6, add(6)(p22), add(6)(p21), add(7)(p13) [4], del(7)(p13), i(8)(q10), der(8;13)(q10;q10), add(9)(p22), der(9;13)(q10;q10), del(10)(p11), der(10;13)(q10; q10), +der(10,13)(q10;q10) [3], add(11)(q23), der(11)add(11)(p11), add(11)(q23), del(11)(q12), +del(11)(q12), del(12)(q11q14), +del(12)(q11q14) [6], der(?)t(?;13)(?;q11), −14, −14, −15, −15, −17, del(17)(p12), del(17)(p11), −18,‐19, +20, +20, add(21)(p11), add(21)(q11), +22, +22, +mar, +mar, +mar [25].

The chromosomal alterations observed were as follows: An additional long arm of chromosome 1 (1q), a deletion in the short arm of chromosome 2 (2p), partial deletions in 3p and also 3q, a deletion and extension in 4p, an extension in 5p, loss of chromosome 6, extension of both short arms of chromosome 6, and extension of the short arm of chromosome 7 (in four mitoses). Moreover, we observed a deletion in 7p, an isochromosome in 8q, an unbalanced whole‐arm translocation of the long arms of (8;13), extension of 9p, unbalanced whole‐arm translocation of the long arms of (9;13), deletion in 10p, unbalanced whole‐arm translocation of the long arms of (10;13), additionally present unbalanced whole‐arm translocation of the long arms of (10;13) (in three mitoses), extension of 11q, altered chromosome 11 with extension in 11q and additional alteration in 11p, deletions in 11q, and interstitial deletion in 12q also duplicated in 6 mitoses. Furthermore, we observed an alteration in chromosome 13, loss of chromosomes 14 and 15, duplicated deletion in 17p, loss of the third chromosomes 17, 18, and 19, two additional chromosomes 20 and 22, duplicated extension in 21p, and three additional marker chromosomes (Figure 3).

We assume that all of these changes are clonal aberrations. Although many alterations were detected, we were only able to identify three alterations that were not present in all mitoses. Therefore, we conclude that the cell line NPC/HK1 is stable and cytogenetically distinct.

## 4 Discussion

Human NPC cell lines are widely accessible in vitro model systems that are easy to manage and offer an abundant supply of material. However, many of these cell lines have been shown to be misidentified [1, 2, 3]. It is concerning that the number of publications using misidentified NPC cell lines is continuously increasing [1].

The characterization of the NPC/HK1 cell line provides significant insights into the genetic landscape of NPC. Derived from a tumor tissue explant [4], NPC/HK1 has been confirmed as a stable, EBV‐negative cell line with distinct phenotypic characteristics that have remained consistent over four decades. This stability enhances its utility as an experimental tool in NPC research, particularly in studies addressing the molecular mechanisms underlying this malignancy.

Our STR profiling results affirm the unique identity of NPC/HK1, distinguishing it from commonly misidentified cell lines such as HeLa and others purported to be of NPC origin [1, 2, 3]. The confirmation of its genetic identity is crucial given the prevalence of contamination issues within established cell lines.

The karyotyping analysis revealed a complex near‐triploid karyotype with numerous chromosomal alterations in NPC/HK1. These changes, such as deletions, extensions, and translocations across various chromosomes, indicate that NPC/HK1 exhibits significant genomic instability. Both numerical genomic instability (gain/loss of chromosomes and extra sets of chromosomes) and structural genomic instability (deletion, amplification, inversion, and translocation) are common in many cancer types [18]. The presence of clonal aberrations suggests that these genetic alterations likely contribute to the tumorigenicity of this cell line. Interestingly, while several chromosomal abnormalities were identified, only a few were absent in all mitoses analyzed, indicating that NPC/HK1 maintains some cytogenetic stability despite its complex karyotype. It should also be noted that the identified chromosomal alterations are different from those found in cell lines C666‐1 and C17, for which initial genetic characteristics are established [11, 19].

Surprisingly, compared to other established NPC lines such as C666‐1, C17, and NP69SV40T, NPC/HK1 tested negative for EBV infection. This finding raises questions about the role of EBV in NPC pathogenesis and emphasizes the importance of using EBV‐negative models to study alternative oncogenic pathways. The high viral loads seen in other cultured NPC lines may indicate different biological behaviors or adaptations compared to those seen in NPC/HK1.

Compared to many other NPC cell lines such as HONE‐1, CNE‐1, CNE‐2, NPC‐TW01, SUNE1, SUNE2, 6‐10B, 5‐8F, and HNE‐1, the cell line NPC/HK1 has a unique genetic profile. This makes NPC/HK1 an essential model for exploring therapeutic strategies and understanding the underlying biology of this malignancy. Further studies are needed to uncover the functional implications of the identified chromosomal alterations and their potential roles in the development and progression of NPC.

## 5 Limitations

While our study provides valuable insights into the genetic characteristics of the NPC/HK1 cell line, several limitations should be acknowledged. First, the karyotyping analysis was based on a limited number of mitoses, which may not fully represent the genetic variability present in the entire cell population. Additionally, while we confirmed the identity of NPC/HK1 through STR profiling, it is important to note that cell lines can undergo genetic drift over time due to prolonged culture conditions, which may modify the defined STR profile and affect their stability and relevance for research. Furthermore, although we have confirmed that NPC/HK1 is EBV‐negative, the functional implications of this characteristic in relation to NPC biology remain to be explored. It is well accepted that the quantification of EBV DNA is a complex issue [20, 21]. The system we used to quantify the viral load in the cell supernatants of the NPC line is designed to quantify EBV DNA in human blood samples, which might result in incorrect absolute values. It should also be noted that even with authentication and genetic characterization, a cell line may not accurately reflect its original tissue or diseases. This is particularly relevant in the case of NPC/HK1, which has lost its EBV expression.

## 6 Conclusions

In this study, we characterized the NPC/HK1 cell line, which was derived from a poorly differentiated squamous carcinoma of a 60‐year‐old Chinese male. Our findings confirm that NPC/HK1 is a stable, EBV‐negative cell line with distinct genetic and phenotypic characteristics that differentiate it from commonly misidentified NPC lines. Through STR profiling, we validated the identity of NPC/HK1, demonstrating its unique profile and confirming its utility for future NPC research. The karyotyping analysis revealed a complex near‐triploid karyotype with significant chromosomal alterations, suggesting that these changes are clonal aberrations inherent to this cell line.

## Author Contributions

**Anna Makowska:** conceptualization (equal), investigation (equal), project administration (supporting), writing – review and editing (supporting). **Udo Kontny:** resources (supporting), writing – review and editing (supporting). **Josef van Helden:** investigation (supporting). **Barbara Hildebrandt:** data curation (equal), investigation (equal), visualization (supporting). **Herdit M. Schüler:** data curation (supporting). **Ralf Weiskirchen:** conceptualization (equal), data curation (equal), investigation (lead), project administration (lead), resources (lead), supervision (lead), visualization (lead), writing – original draft (lead), writing – review and editing (lead).

## 7 Conflicts of Interest

The authors declare no conflicts of interest.

## Supporting information


Figures S1‐S2.

## Data Availability

Additional data that support the findings of this study are available in the Supporting Information of this article.
