# Supplementary material for: Genetic Characterization of the Immortalized Human Nasopharyngeal Carcinoma Cell Line NPC/HK1
Source: Cancer Med. 2025 Feb 4;14(3):e70422. doi: 10.1002/cam4.70422 (PMC11794828; doi:10.1002/cam4.70422)
Supplement: Supplementary file 1 — Figures S1‐S2. [file CAM4-14-e70422-s001.docx]

**Supplementary Figures**

**Genetic characterization of the immortalized human nasopharyngeal carcinoma cell line NPC/HK1**

**Anna Makowska^1^, Udo Kontny^1^, Josef van Helden^2^, Barbara Hildebrandt^3^, Herdit M. Schüler^3^, Ralf Weiskirchen^2^**


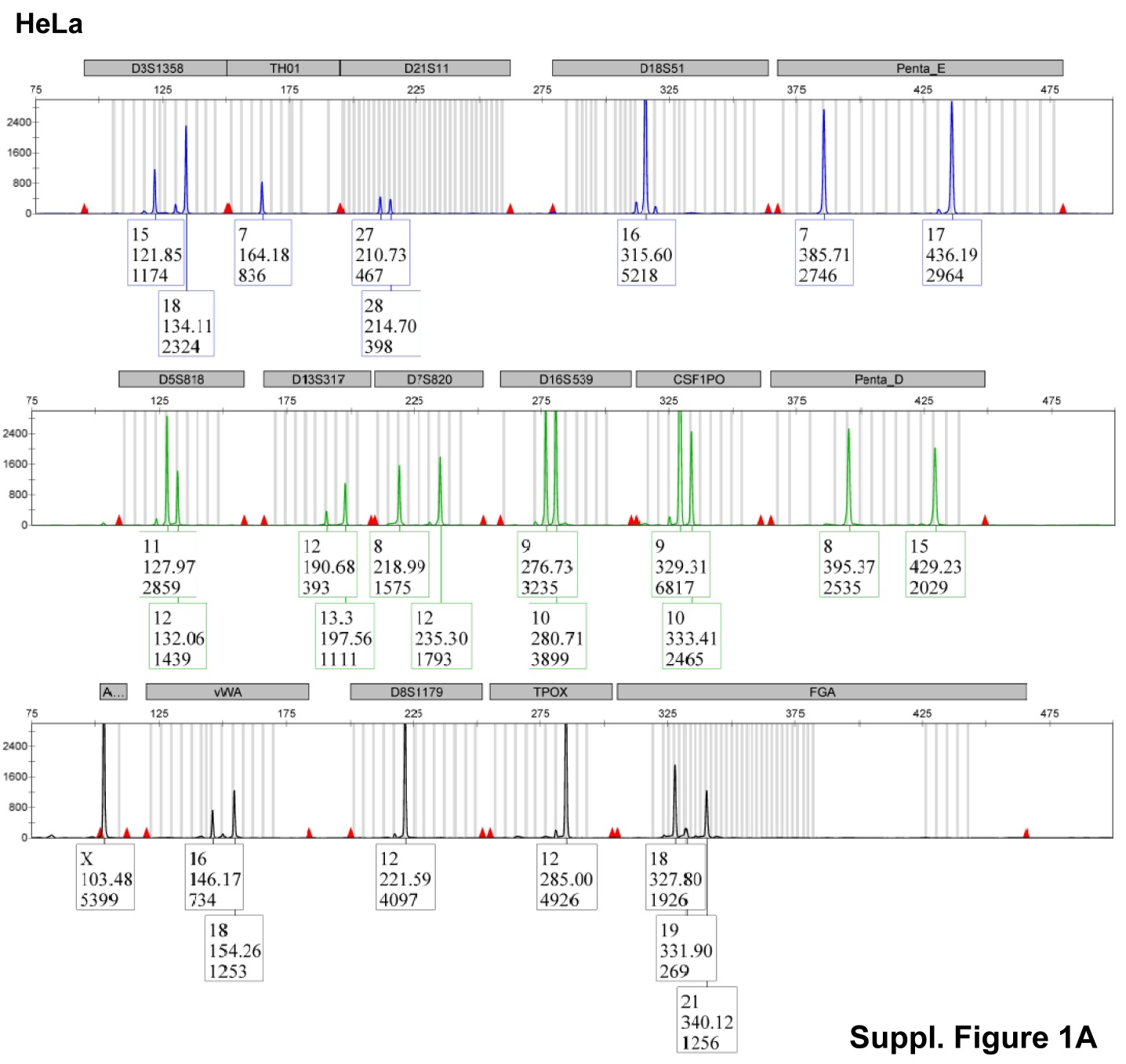


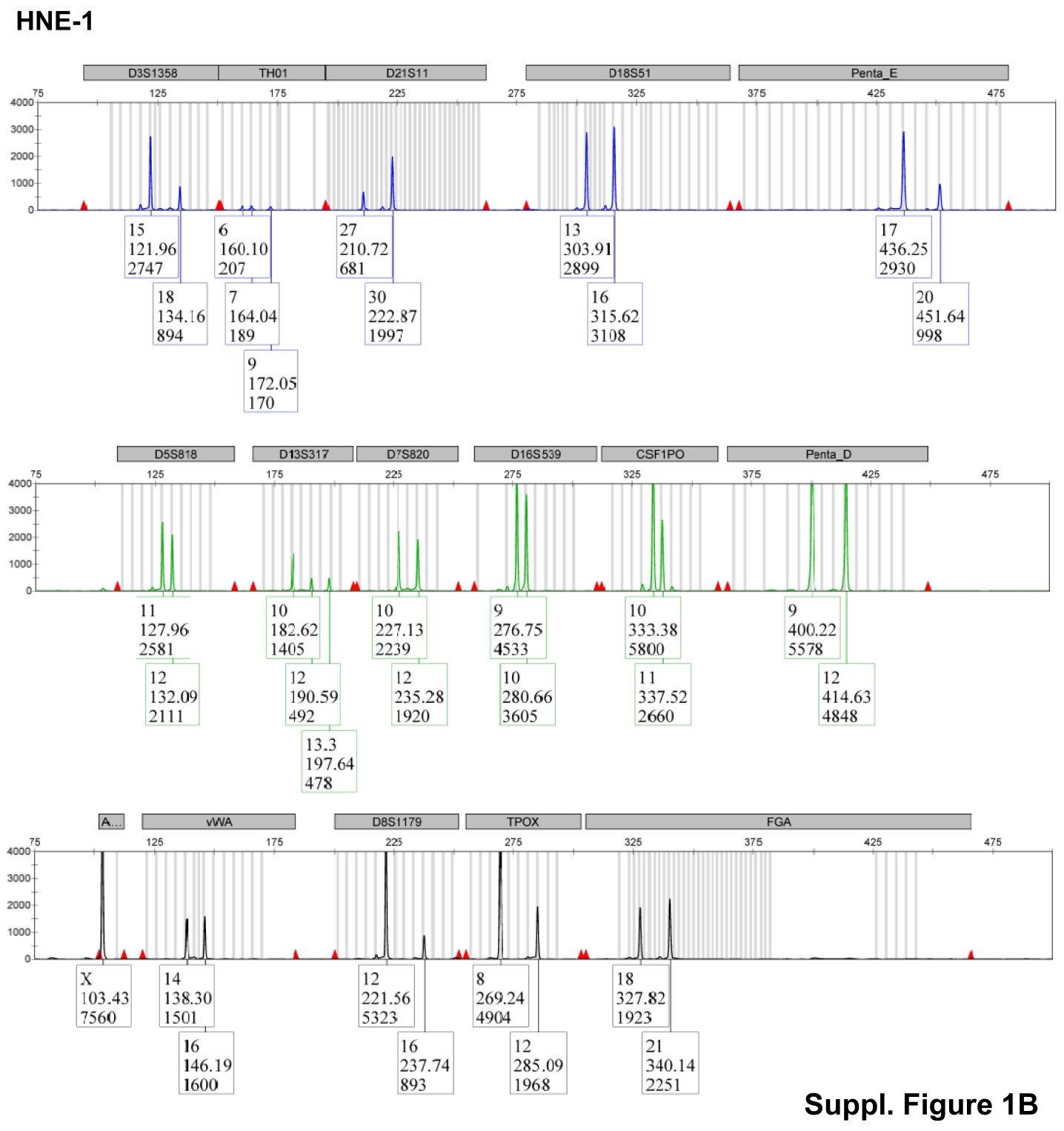


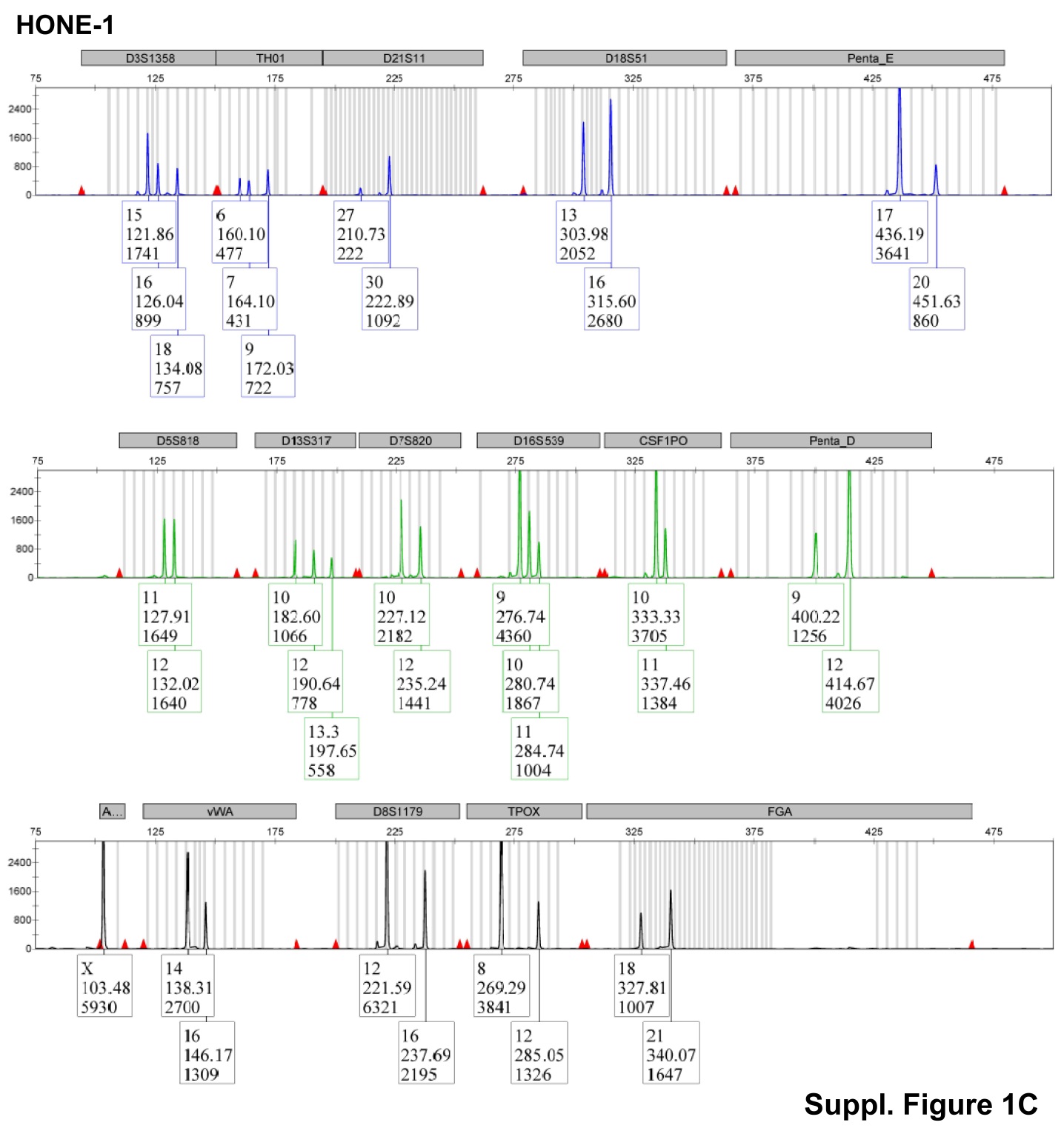

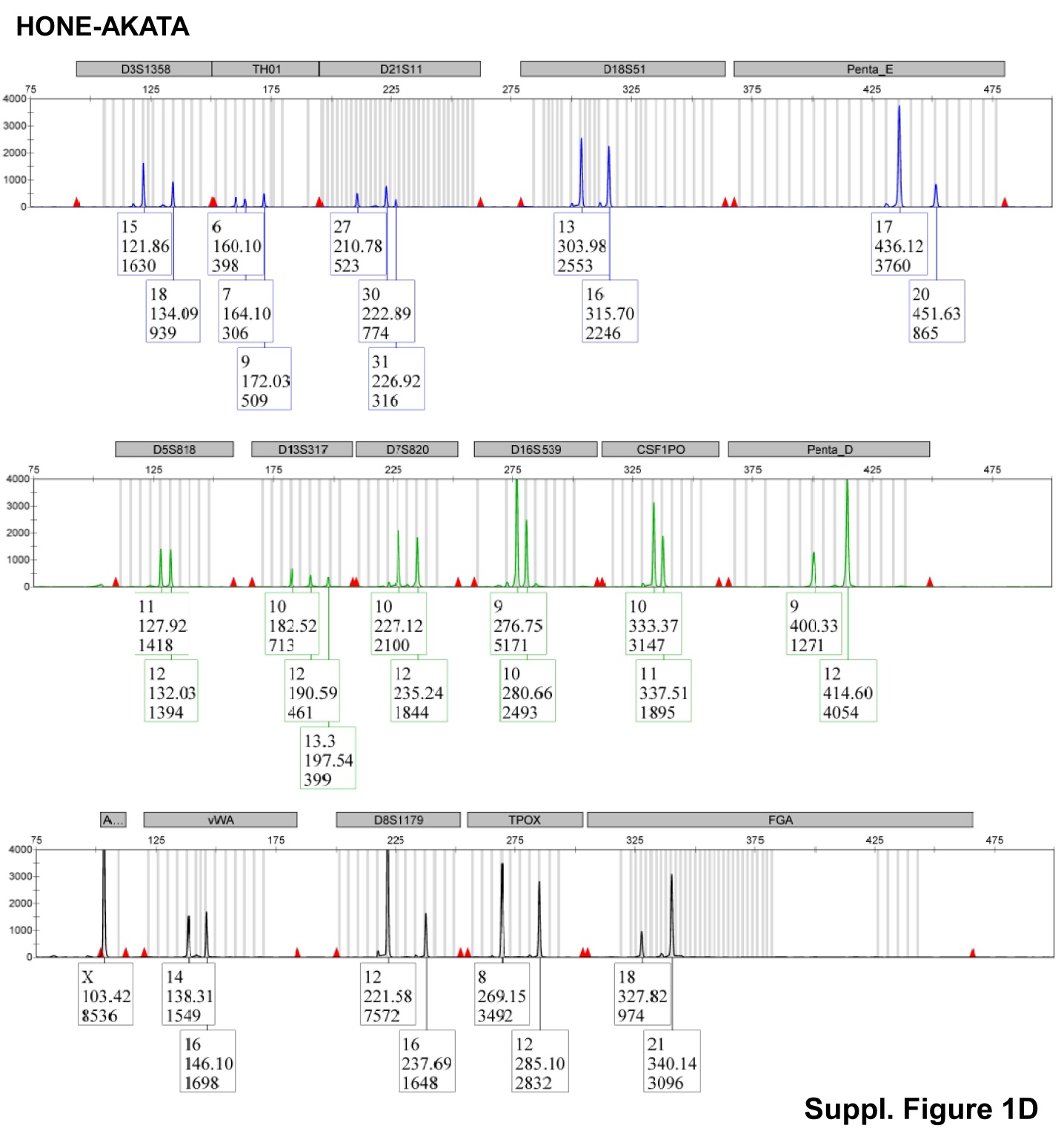


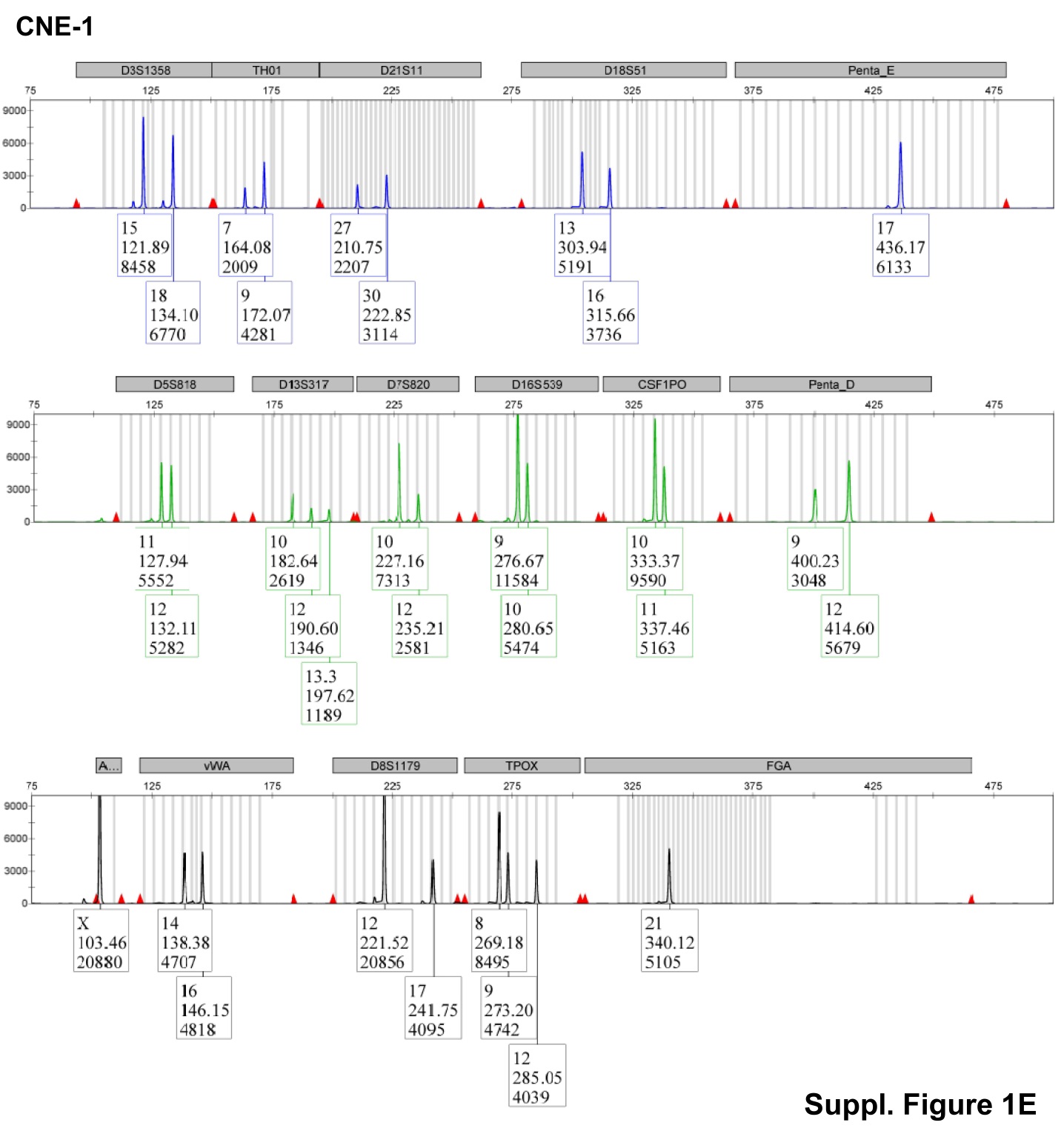


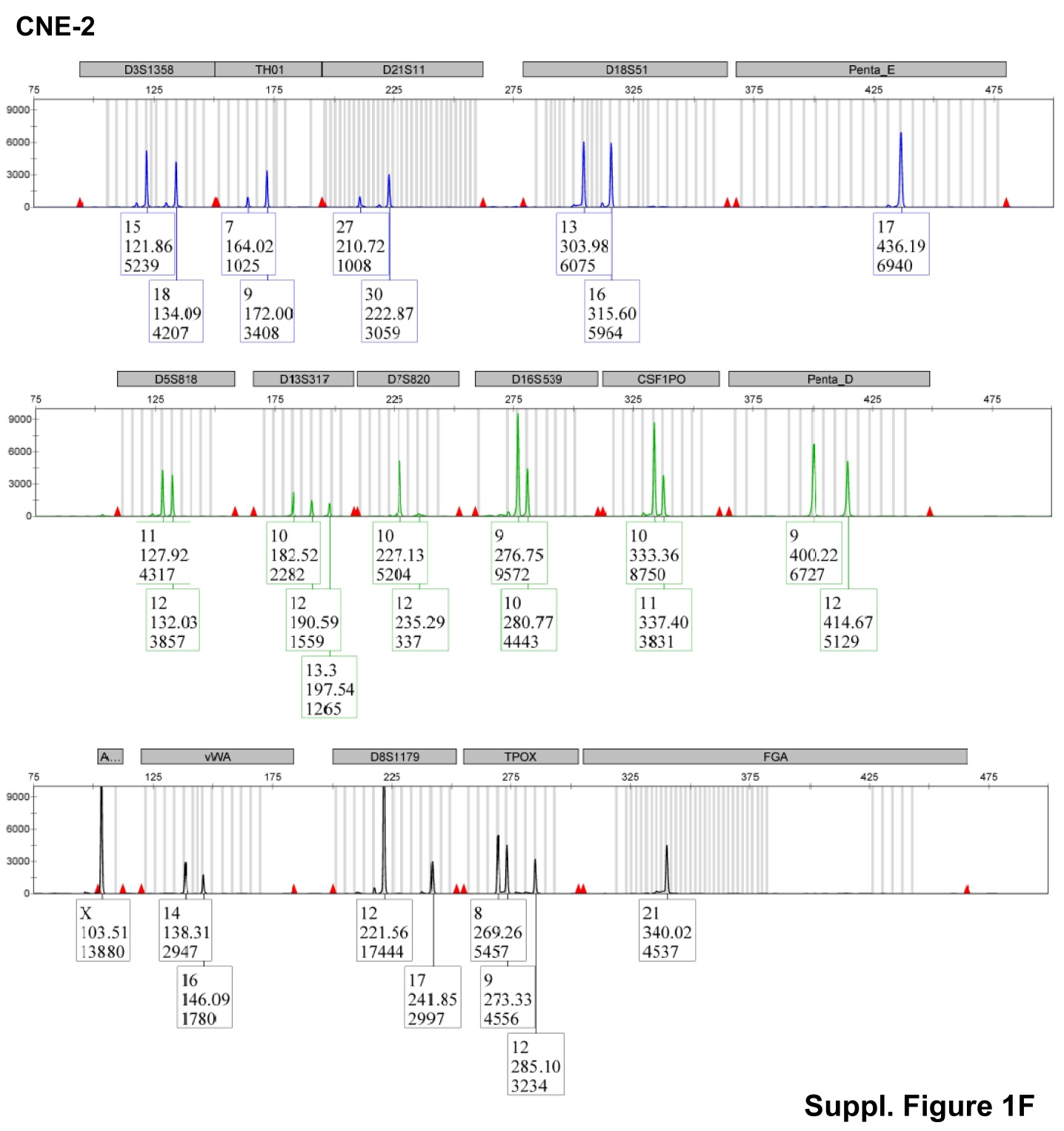


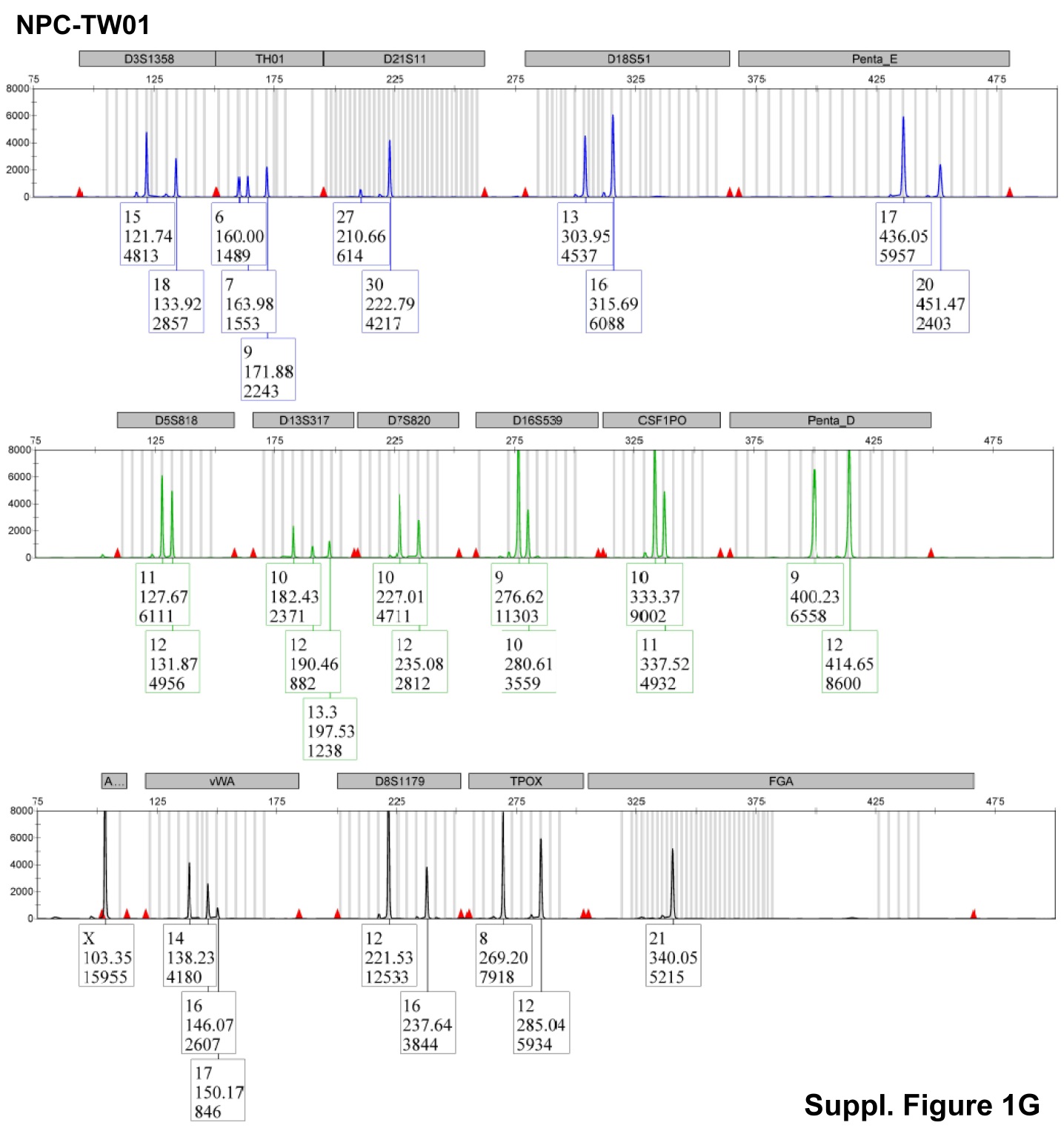


**Supplementary Figure 1: Short tandem repeat profiles. STR profiles depicted were derived from cell lines (A)** HeLa, **(B)** HNE-1, **(C)** HONE-1, **(D)** HONE-1/AKATA, **(E)** CNE-1, **(F)** CNE-2, and **(G)** NPC-TW01.


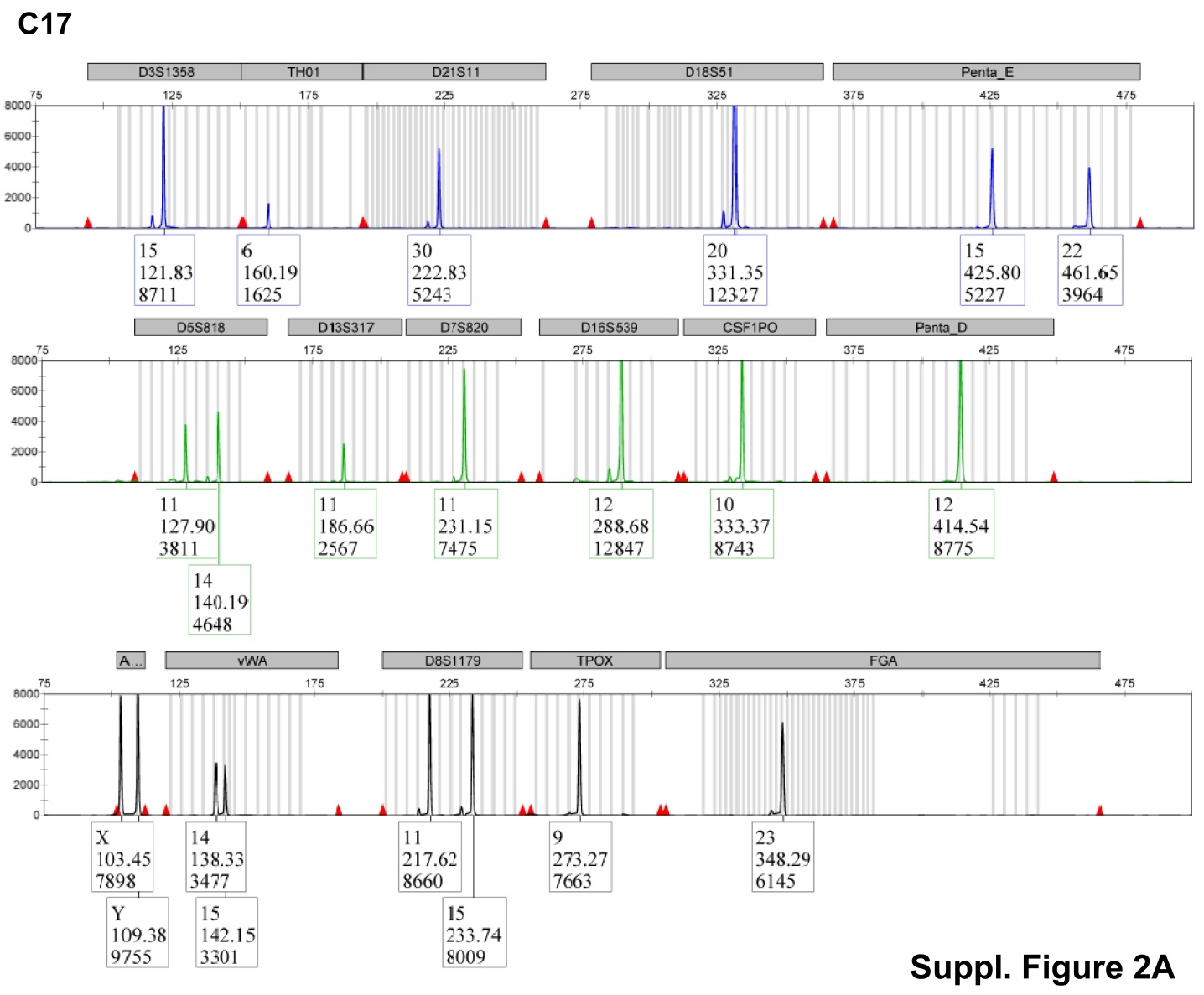


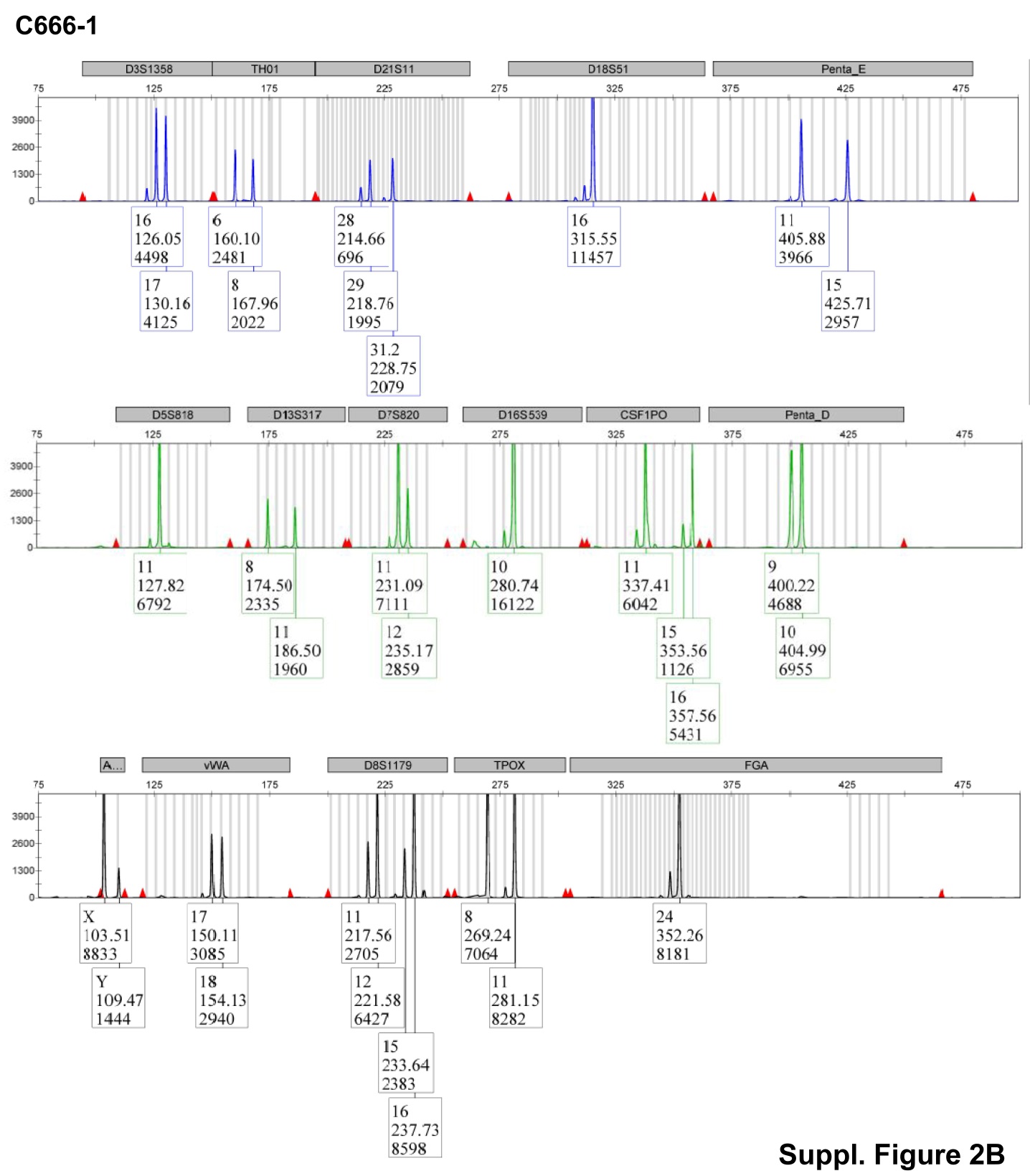


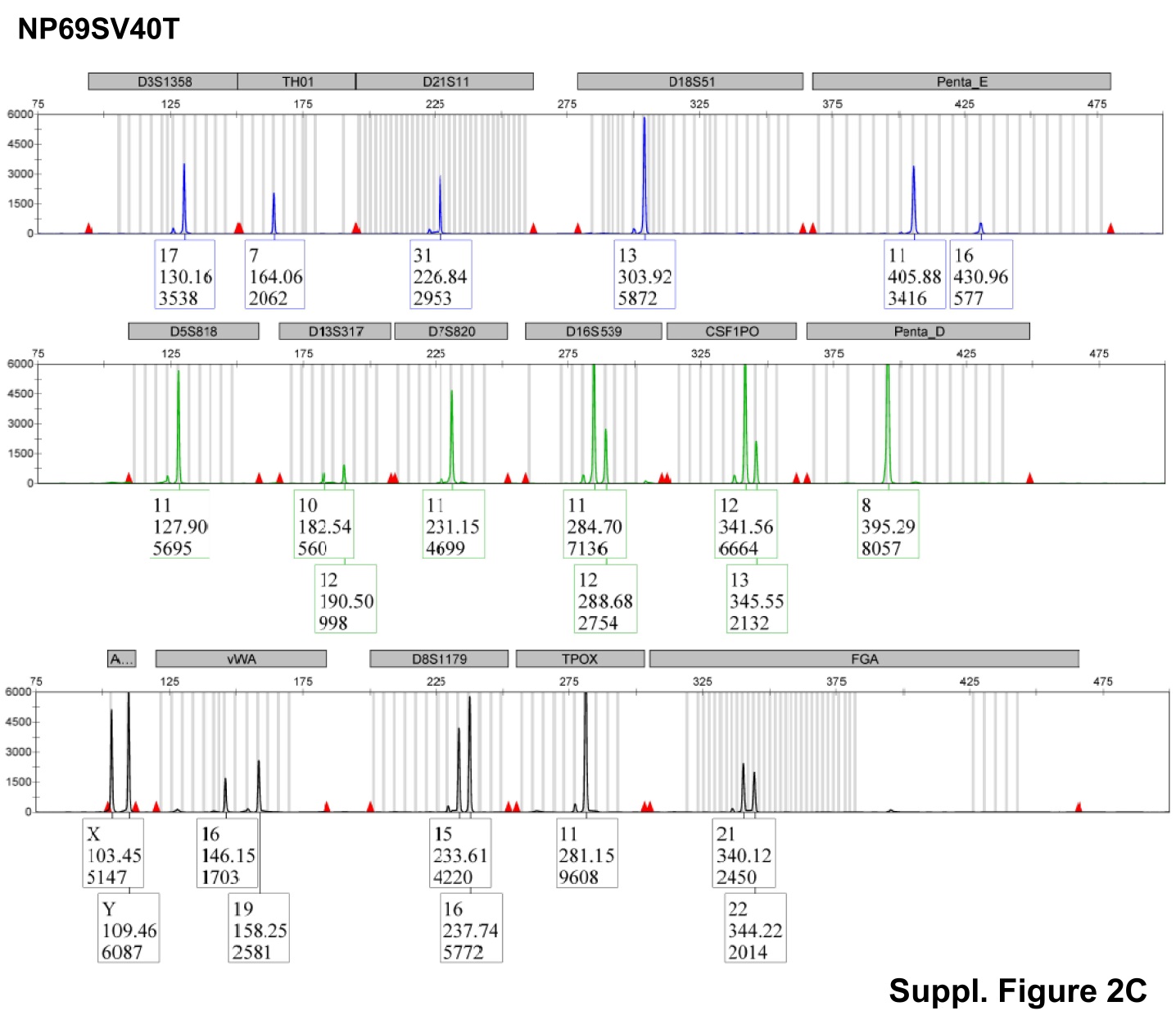


**Supplementary Figure 2: Short tandem repeat profiles from NPC lines.** The depicted electropherograms were derived from cell lines **(A)** C17, **(B)** C666-1, and **(C)** NP69SV40T.
